# Betamethasone (Veterinary Medicinal Products)

**DOI:** 10.14252/foodsafetyfscj.D-20-00016

**Published:** 2020-06-26

**Authors:** Food Safety Commission of Japan

## Abstract

The Food Safety Commission of Japan (FSCJ) conducted a risk assessment of betamethasone (CAS No. 37-44-9), a synthetic adrenocortical hormone, based on the documents including assessment reports from the European Medicines Evaluation Agency (EMEA). Among results of various studies, a no-observed-adverse-effect level (NOAEL) of betamethasone (as betamethasone dipropionate) was shown as 0.02 mg/kg bw per day in a fertility and early embryonic development study in rats. FSCJ concluded that it is appropriate to set an acceptable daily intake (ADI) of betamethasone by applying the same ADI as dexamethasone. The ADI for dexamethasone at 0.01 μg/kg bw per day (0.00001 mg/kg bw/day) was specified based on the NOAEL of 0.001 mg/kg bw per day of the endocrine toxicity study in rats^1)^. Consequently, FSCJ specified the ADI for betamethasone at 0.01 μg/kg bw per day.

## Conclusion in Brief

The Food Safety Commission of Japan (FSCJ) conducted a risk assessment of betamethasone (CAS No. 37-44-9), a synthetic adrenocortical hormone, based on the documents including assessment reports from the European Medicines Evaluation Agency (EMEA).

The data used in the assessment include Absorption, Distribution, Metabolism, Excretion (ADME) (rats, rabbits, cattle and humans), residues (cattle and pigs), genotoxicity, acute toxicity (mice, rats and dogs), subacute toxicity (rats, dogs and monkeys), carcinogenicity (mice and rats), as well as reproductive and developmental toxicity (mice, rats and rabbits). Other data include pharmacological effects, immunotoxicity *in vitro*, tyrosine aminotransferase (TAT) activity in rat liver, titer comparison for pharmacological effects among major corticosteroids, and observation in humans.

In assays of *in vitro* gene mutation, betamethasone showed negative results for bacteria and mammalian cells. A positive result was obtained in an *in vitro* chromosomal aberration assay, whereas an *in vivo* micronucleus test was negative. These data indicated no genotoxicity relevant to human health. It was thus able to specify the acceptable daily intake (ADI) for betamethasone.

Major adverse effects of betamethasone including suppressed body weight as well as atrophy of thymus and spleen were attributable to glucocorticoid activity of betamethasone.

In a 104-week carcinogenicity study in rats by gavage, none of effects of betamethasone was observed on tumor incidence. Although the route of administration was not oral, no tumorigenicity was observed on a 104-week dermal carcinogenicity study in mice. Thus, betamethasone was judged to be non-carcinogenic.

Malformations, including cleft palate observed in developmental toxicity studies in rats and rabbits, are possible to relate to the glucocorticoid activity. None of the adverse effect was detected after the subcutaneous injection of 0.003 mg/kg bw or less per day in a developmental study in rabbits. It is reasonably assumed that no-observed-effect level (NOEL) of the oral administration was higher than the level of subcutaneous route.

Among results of various studies, a no-observed-adverse-effect level (NOAEL) of betamethasone (as betamethasone dipropionate) was shown as 0.02 mg/kg bw per day in a fertility and early embryonic development study in rats. Toxicities including suppressed body weight, decrease in feed consumption and reduction of the thymus weight were, however, observed at this dose in a 4-week subacute toxicity study and in the 104-week carcinogenicity study in rats. Thus FSCJ considered it not appropriate to set an ADI based on this NOAEL, 0.02 mg/kg bw per day.

NOEL of 0.004 mg/kg bw per day was identified based on increased TAT activity in rat liver. However, FSCJ judged this endpoint not appropriate for establishment of an ADI. Increased TAT activity is rather physiological but not adverse reaction to glucocorticoids.

FSCJ recognizes that betamethasone has similar toxicological properties and equivalent glucocorticoid activity to dexamethasone, an epimer, which was evaluated by FSCJ^1)^. The adverse effects of dexamethasone were attributable to glucocorticoid actions of dexamethasone. Taken together, FSCJ concluded that it is appropriate to set an ADI of betamethasone by applying the same ADI as dexamethasone. The ADI for dexamethasone at 0.01 μg/kg bw per day (0.00001 mg/kg bw/day) was specified based on the NOAEL of 0.001 mg/kg bw per day of the endocrine toxicity study in rats^1)^.

Consequently, FSCJ specified the ADI for betamethasone at 0.01 μg/kg bw per day.

**Table 1.** *Levels relevant of toxicological evaluation of betamethasone*

Species	Study	Dose (mg/kg bw/day)	NOAEL (mg/kg bw/day)
Rat	Pharmacology	dose unknown (oral)	0.004 (NOEL) TAT activity in liver
	4-week acute toxicity study	0, 0.02, 0.06, 0.2 (oral)^a^	0.02 (LOAEL) Suppressed body weight, decrease in feed consumption and thymus weight
	13-week acute toxicity study	0, 0.02, 0.06, 0.2 (oral)^a^	0.02 (LOAEL) Suppressed body weight, decrease in thymus and spleen weight
	28-week ~ 9-month acute toxicity study	0, 0.024 ~ 3 (oral) 0, 0.24 ~ 30 (oral)^c^	-
	104-week carcinogenicity study	0, 0.02, 0.06, 0.2 (oral)^a^	0.02 (LOAEL) Loss of fur, decrease in WBC and increase in mat cells in the mesenteric lymph node
	Administration before mating and in early pregnancy (the 1^st^ study)	M: 0, 0.02, 0.06, 0.2 (oral)^a^ F: 0, 0.1, 0.3, 1 (oral)^a^	M: 0.02 (NOAEL) Suppressed body weight as well as atrophy of thymus and spleen M Fertility: 0.2 (NOAEL) No effect of administration F: 0.1 (LOAEL) Suppressed body weight and decrease in thymus weight F Fertility and early embryonic development: 1 (NOAEL) No effect of administration
	Administration beforemating and in earlypregnancy(the 2^nd^ study)	0, 0.01, 0.1, 1 (subcutaneous)^d^	-
	Administration during the perinatal and lactating period (the 1^st^ study)	0, 0.1, 0.3, 1 (oral)	Maternal and neonatal: 0.1 (LOAEL)
	Administration during the perinatal and lactating period (the 2^nd^ study)	0, 0.004, 0.04, 0.4 (subcutaneous)^d^	
	Developmental toxicity (the 2^nd^ study)	0, 0.05, 0.4, 3.2 (subcutaneous)^d^	
	Pharmacology	dose unknown (oral)	0.004 (NOEL) TAT activity in liver
Dog	6-week subacute toxicity	1 (oral) 0, 0.5, 1, 2 (oral)^c^	0.5^c^ (LOAEL)
Monkey	12-month repeated dose toxicity study	0, 0.2, 0.4, 0.8, 2 (oral)	
Rabbit	Developmental toxicity	0, 0.0001, 0.001, 0.003, 0.01 (subcutaneous)^d^	
Basis for setting the ADI			Applying the same ADI of dexamethasone
The critical study for setting the ADI			Studies for endocrine effect in rats (dexamethasone)
ADI (mg/kg bw/day)			0.00001

M, Male; F, Female; ADI, Acceptable daily intake; LOAEL, Lowest-observed-adverse-effect level; NOAEL, No-observed-adverse-effect level; NOEL; No-observed-effect level; TAT, Tyrosine aminotransferase; -, NOAEL could not be specified, and toxicity was not listed in the table due to testing details unavailable, subcutaneous injection or transdermal administration

a, As Betamethasone dipropionate

b, As Beclomethasone

c, As Betamethasone valerate ester

d, As Betamethasone butyrate ester propionate
